# pHLIP(Var7)-P1AP suppresses tumor cell proliferation in MDA-MB-231 triple-negative breast cancer by targeting protease activated receptor 1

**DOI:** 10.1007/s10549-020-05560-2

**Published:** 2020-02-07

**Authors:** MingMing Yu, YueHua Chen, ZhenGuang Wang, XiaoDong Ding

**Affiliations:** 1 Department of Nuclear Medicine, The Affiliated Hospital of Qingdao University, No. 59, Haier Rd., Qingdao, 266100 China; 2 Intensive Care Unit, The Affiliated Hospital of Qingdao University, Qingdao, China; 3 Department of Neurosurgery, The Affiliated Hospital of Qingdao University, Qingdao, China

**Keywords:** pH (low) insertion peptides (pHLIP), Protease-activated receptor 1 (PAR1), Triple-negative breast cancer (TNBC), Cell proliferation

## Abstract

**Purpose:**

Protease-activated receptor 1 (PAR1) is a signaling protein ubiquitously present on the surface of tumor cells, and its homologous protein fragment, PAR1-activating peptide (P1AP), can inhibit protein signal transduction of PAR1/G in tumor cells. pH (Low) insertion peptide (pHLIP) can target the acidic tumor microenvironment (TME) and can be used as an excellent carrier to deliver P1AP to tumor cells for therapeutic purposes.

**Methods:**

PAR1 expression on the surface of MDA-MB-231 cells and human MCF10A mammary epithelial cells was observed. The binding between fluorescent-labeled pHLIP(Var7)-P1AP and MDA-MB-231 cells under different pH values was analyzed. The effect of pHLIP(Var7)-P1AP on the proliferation of MDA-MB-231 cells was analyzed under the conditions of pH 7.4 and 6.0.

**Results:**

PAR1 was highly expressed on the surface of MDA-MB-231 cells. In an acidic environment (pH 6.0 and 5.0), fluorescent-labeled pHLIP(Var7)-P1AP and MDA-MB-231 cells had a high binding ability, and the binding ability increased with the decrease in pH. In an acidic environment (pH 6.0), pHLIP(Var7)-P1AP significantly inhibited MDA-MB-231 cell proliferation. With 0.5 μg, 1 μg, 2 μg, 4 μg, and 8 μg of pHLIP(Var7)-P1AP, the cell proliferation inhibition rates were 3.39%, 5.27%, 14.29%, 22.14%, and 35.69%, respectively.

**Conclusion:**

PAR1 was highly expressed on the surface of MDA-MB-231 cells. pHLIP(Var7)-P1AP can effectively target MDA-MB-231 cells in an acidic environment and inhibit the growth of MDA-MB-231 cells by inhibiting the signal transduction of PAR1/G protein.

## Introduction

Triple-negative breast cancer (TNBC) refers to breast cancer tumor cells that do not express estrogen receptor (ER), progesterone receptor (PR), and human epidermal growth factor receptor 2 (Her-2). It accounts for 15–20% of breast cancer cases. TNBC has some clinical features, such as strong invasiveness, fast progression, and poor prognosis. The endocrine therapies and targeted therapies commonly used for the treatment of breast cancer are ineffective for TNBC. Therefore, the development of new therapeutic drugs targeting TNBC needs to be addressed in an urgent manner to improve the treatment strategies [1].

Protease-activated receptor 1 (PAR1) participates a variety of tumor invasion and metastasis processes and is a potential target for tumor treatment [2]. Pepducin PZ-128 is a palmitoylated peptide composed of a lipid and a PAR1 homologous protein fragment, PAR1-activating peptide (P1AP, KKSRALF). It has been shown that PZ-128 can inhibit PAR1/G protein signal transduction in tumor cells by targeting the third intracellular loop of PAR1 [2]. pH (Low) insertion peptide (pHLIP) family members are a novel type of vector that can target the acidic tumor microenvironment (TME) [3, 4]. The molecular mechanism of targeting is based on pHLIP insertion into the tumor cell membrane in a TME with low pH and the formation of a pH-dependent transmembrane α-helices [5]. pHLIP inserts its C-terminus across the cell membrane into tumor cells, where therapeutic molecules can be delivered.

In this study, the C-terminus of pHLIP(Var7) and the N-terminus of P1AP were linked by a disulfide bond, and pHLIP(Var7)-P1AP was obtained. pHLIP(Var7) inserted P1AP, which was linked to the C-terminus, into MDA-MB-231 cells after cleavage of the disulfide bond. The PAR1/G protein signal transduction was inhibited by targeting the intracellular loop of PAR1, achieving the purpose of treating MDA-MB-231 TNBC.

## Materials and methods

### Materials

PAR1 rabbit monoclonal antibody was purchased from Affinity Biosciences. Cy3-conjugated goat anti-rabbit IgG was purchased from CWBIO. 4′,6-Diamidino-2-phenylindole (DAPI) was purchased from KeyGen Biotech. 3-(4,5-dimethylthiazol-2-yl)-2,5-diphenyltetrazolium bromide (MTT) was purchased from Sigma-Aldrich.

### Design, synthesis, and purification of (FITC) pHLIP(Var7)-P1AP

pHLIP(Var7)Cys and CysP1AP sequences were prepared by solid phase polypeptide synthesis. (pHLIP(Var7)Cys: AEEQNPWARYLEWLFPTETLLLELC; CysP1AP: CKKSRALF). pHLIP(Var7)Cys and CysP1AP were linked by disulfide bond to obtain pHLIP(Var7)-P1AP. The N-terminus of pHLIP(Var7)-P1AP was labeled with fluorescein isothiocyanate (FITC), and the C-terminus was modified by amidation to eventually obtain (FITC)pHLIP(Var7)-P1AP: (FITC)AEEQNPWARYLEWLFPTETLLLELC-CKKSRALF-NH_2_. Peptides were purified via reversed-phase high-performance liquid chromatography (RP-HPLC) (Gemini-NX 10 μm, C18, 100 A, 4.6 × 250 mm; flow rate 1.0 mL/min; phase A: 0.1% trifluoroacetic acid in 100% acetonitrile; phase B: 0.1% trifluoroacetic acid in 100% water; gradient, 30 min from 28/72 A/B to 100/0 A/B). The purity of the peptide was determined by RP-HPLC as provided, and its identity was confirmed via mass spectrometry.

### Cell culture and PAR1 expression on the cell surface

The human breast cancer cell line MDA-MB-231 and normal human breast epithelial cell line MCF-10A were purchased from the Cell Research Institute of the Chinese Academy of Sciences. MDA-MB-231 cells were cultured in L-15 medium containing 10% fetal bovine serum (FSB) at 37 °C without CO_2_. MCF-10A cells were cultured in Dulbecco's Modified Eagle Medium (DMEM)/F12 (1:1) + horse serum (5%) + insulin (10 µg/mL) + epidermal growth factor (20 ng/mL) + cholera toxin (100 ng/mL) + hydrocortisone (0.5 µg/mL) at 37 °C with 5% CO_2_.

#### PAR1 expression on the surface of MDA-MB-231 and MCF-10A cells

The cultured cells were washed with phosphate-buffered saline (PBS) three times, fixed in 4% paraformaldehyde for 15 min, and washed with PBS three times. The cells were permeabilized with 0.5% Triton X-100 (prepared with PBS) for 20 min at room temperature. The cells were washed with PBS three times, and 5% bovine serum albumin (BSA) was added; then, the cells were incubated for 30 min at 37 °C. The blocking buffer was aspirated by pipette. Adequate amounts of diluted PAR1 rabbit monoclonal antibody (1:200) was added to the culture dish, and the cells were incubated at 37 °C for 3 h. The cells were washed with PBS three times, diluted fluorescence Cy3-conjugated goat anti-rabbit IgG (1:200) was added dropwise, and the cells were incubated at 37 °C for 30 min. The cells were washed with PBS three times. DAPI was added dropwise, and the cells were incubated in the dark for 5 min to stain the cell nuclei. Excess DAPI was washed away with PBS. The cells in the culture dish were blocked with 50% glycerol, and images were obtained under a fluorescence microscope (OLYMPUS).

### Analysis of (FITC)pHLIP(Var7)-P1AP binding to MDA-MB-231 cells

MDA-MB-231 cells were inoculated into 24-well plates (5.0 × 10^4^ cells/well). After the cells attached to the plates, the culture medium was removed, and phosphate buffer (25 mmol/L, pH 7.4) was used to wash the cells. The cells were divided into three groups, and L-15 medium (pH of 7.4, 6.0, or 5.0) was added to the wells, and the cells were incubated overnight at 37 ℃ without CO_2_. A total of 30 μL of 100 nM (FITC)pHLIP(Var7)-P1AP was added to each well, and the plate was placed in a shaking mixer in an incubator for 2 h. All cells were washed five times with the corresponding pH (7.4, 6.0, 5.0)-specific phosphate buffer (25 mmol/L) to remove any unbound probes; then, fluorescence imaging was performed.

### Antiproliferation assay

MDA-MB-231 cells were inoculated into 24-well plates (5.0 × 10^4^ cells/well). After the cells attached to the plates, the culture medium was removed, and phosphate buffer (25 mmol/L, pH 7.4) was used to wash the cells. L-15 medium with a pH of 7.4 or 6.0 was added to the wells, and the cells were incubated overnight at 37 ℃ without CO_2_. Different amounts of pHLIP(Var7)-P1AP (0, 0.5 μg, 1 μg, 2 μg, 4 μg, and 8 μg) were added into the wells, and then, the plate was placed in an incubator for 72 h. All cells were washed five times with the corresponding pH (7.4 or 6.0)-specific phosphate buffer (25 mmol/L) to remove any unbound probes. Cell viability was determined by colorimetric MTT assay. A total of 20 μL of 5 mg/mL MTT solution was added to the treated cells, and the cells were incubated at 37 ℃ for 4 h. The obtained crystals were dissolved in 150 μL of dimethyl sulfoxide (DMSO), and the absorbance was measured at 490 nm using a microplate reader to calculate the cell proliferation inhibition rate.

### Statistical analysis

SPSS 24.0.0.0 (IBM) statistical software was used for data processing. Variables are expressed as the mean (*M*) ± standard deviation (SD). Using one-way ANOVA to compare variables, *p* values less than 0.05 were considered statistically significant.

## Results

### Synthesis and purification of (FITC)pHLIP(Var7)-P1AP

(FITC)pHLIP(Var7)-P1AP was successfully synthesized. pHLIP(Var7)Cys and CysP1AP were prepared by solid phase polypeptide synthesis. pHLIP(Var7)-P1AP was obtained by the formation of a disulfide bond between the two peptides through oxidation. After labeling the N-terminus of pHLIP(Var7)-P1AP with FITC, (FITC)pHLIP(Var7)-P1AP was obtained.

HPLC analysis of the product indicated the formation of several compounds, i.e., a major product (96.8777%, retention time of 11.193 min) accompanied by at least two smaller peaks with retention times of 11.033 min and 11.508 min. The mass spectrometry (ESI–MS) analysis of (FITC)pHLIP(Var7)-P1AP showed three mass peaks with m/z of 753.8 ([M+6H]6+), 904.3 ([M+5H]5+), 1130.2 ([M+4H]4+). The experimentally observed molecular weight (4516.8, 4516.5) correlated well with the theoretical molecular weight (4517.17).

### PAR1 expression on the surface of MDA-MB-231 and MCF-10A cells

**Fig. 1:**
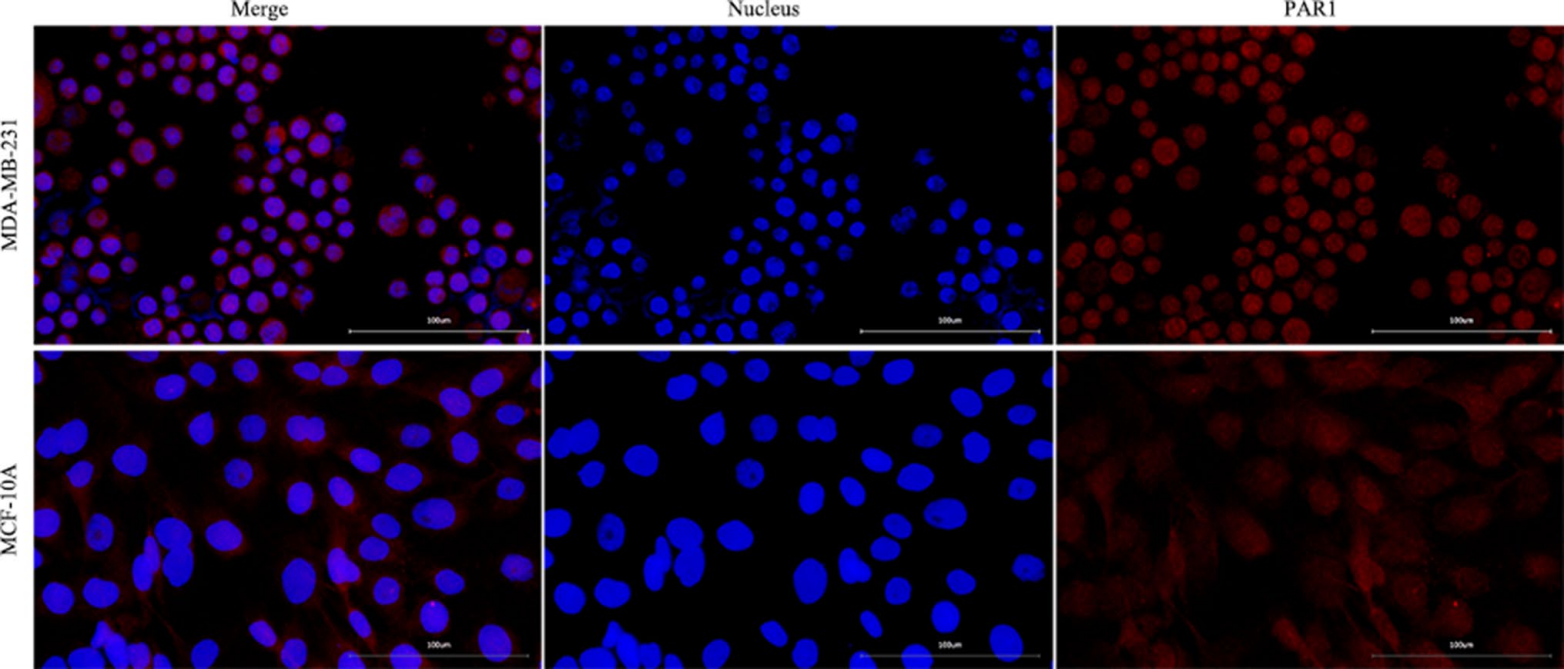
PAR1 expression was positive in MDA-MB-231 cells, and PAR1 expression was negative in MCF-10A cells (400 ×)

PAR1 was highly expressed on MDA-MB-231 cells, and no obvious PAR1 expression was found on MCF-10A cells (Fig. 1)

### Analysis of (FITC)pHLIP(Var7)-P1AP binding to MDA-MB-231 cells

**Fig. 2:**
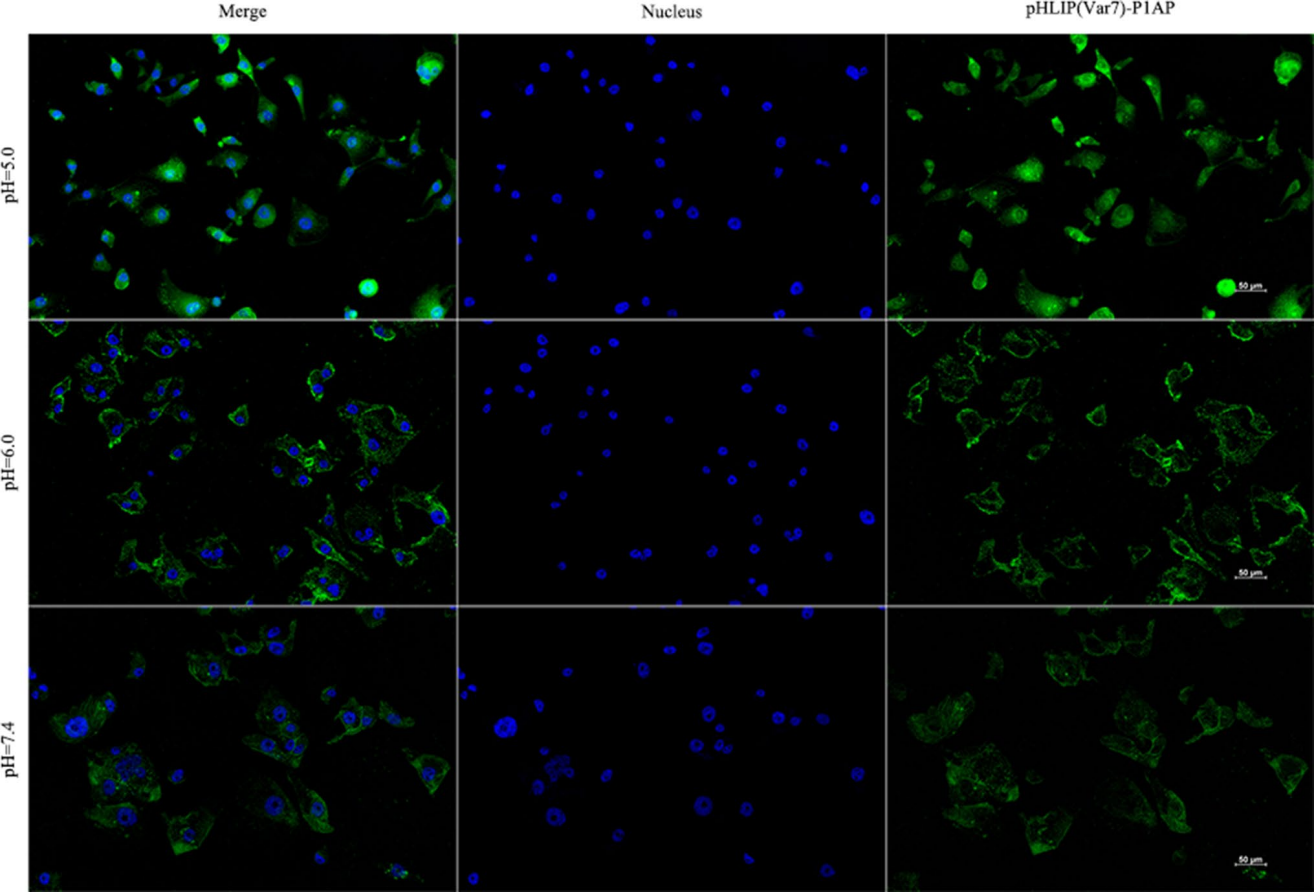
Fluorescence imaging of (FITC)pHLIP(Var7)-P1AP binding to MDA-MB-231 cells (200 ×)

For the acidic pH values (pH 5.0 and 6.0), the probe and MDA-MB-231 cells had a high binding ability; the binding ability at pH 5.0 was higher than that at pH 6.0. At pH 7.4, the probe and MDA-MB-231 cells had only slight binding (Fig. 2).

### Antiproliferation assay

**Fig. 3:**
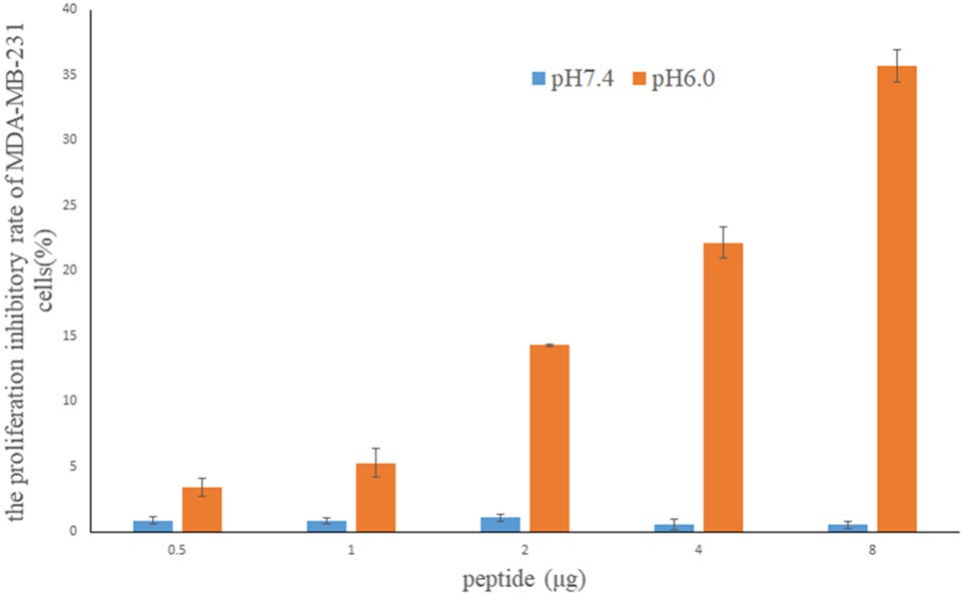
The effect of pHLIP(Var7)-P1AP on MDA-MB-231 cell proliferation under different pH conditions

Compared with pH 7.4, pHLIP(Var7)-P1AP significantly inhibited MDA-MB-231 cell proliferation at pH 6.0 (*p* < 0.05) (Fig. 3). At pH 6.0 with probe doses of 0.5 μg, 1 μg, 2 μg, 4 μg, and 8 μg, the cell proliferation inhibition rates for MDA-MB-231 cells were (3.39 ± 0.7)%, (5.27 ± 1.1)%, (14.29 ± 0.1)%, (22.14 ± 1.2)% and, (35.69 ± 1.2)%, respectively.

## Discussion

TNBC occurs at a young age and is prone to early metastasis. Due to the lack of corresponding receptors, conventional endocrine therapies and targeted therapies are not effective, leading to a research hotspot in the field of breast cancer [1]. PAR1 is a G protein-coupled receptor (GPCR), exists on the surface of a variety of tumor cells and is a potential target for tumor treatments [2]. This study verified that a large amount of PAR1 exists on the surface of TNBC MDA-MB-231 cells, while there is almost no PAR1 expression on the surface of normal human MCF-10A breast cells; therefore, PAR1 is expected to become a potential target for the diagnosis and treatment of MDA-MB-231 TNBC.

The PAR1 inhibitor pepducin PZ-128 (palmitic acid-P1AP) is a class of lipid peptide that can enter cells and inhibit PAR1/G protein signal transduction [2]. PZ-128 is in phase II clinical trials. Currently, it is under development to target the PAR1 on the platelet surface to inhibit platelet function, which is essential for the effective treatment of patients with acute coronary syndrome (ACS) and patients undergoing percutaneous coronary intervention (PCI) [6]. Other studies confirmed that PZ-128 can inhibit the growth and metastasis of breast cancer, lung cancer, and ovarian cancer cells [7]. The exact mechanisms of PZ-128 with respect to GPCR remain unclear, but some researchers have proposed that peptides can directly interact with their homologous receptors, allowing the receptors to have an active or non-active conformation [8]. The effective part of PZ-128 that can inhibit the signal transduction of PAR1/G protein is the P1AP heptapeptide sequence, and the contained palmitic acid helps PZ-128 enter cells. PZ-128 is not able to specifically target tumor cells. If P1AP can be delivered into tumor cells using a tumor-specific carrier instead of palmitic acid, probe entry into normal cells can be minimized and background and adverse effects can be decreased in vivo.

The TME is acidic [9, 10]. The intracellular pH of almost all solid tumors is neutral to alkaline, while the extracellular pH is acidic and can be as low as 6.0 [3, 4, 11]. The mechanisms of an acidic TME include hypoxia-induced anaerobic glycolysis, aerobic glycolysis (Warburg effect), increased CO_2_ production due to uncontrolled cell growth and increased ion pump activity on the cell membrane [9, 11]. An acidic TME is stable and is unaffected by the clonal selection of tumors. Therefore, it is also considered a promising marker for tumor-targeted detection [3, 5]. Research has shown that pHLIP family peptides can target TMEs. This peptide is derived from the bacterial rhodopsin C-helix, originally known as BRC peptide [12]. pHLIP can sense the pH near the cell membrane and insert into the cell membrane spontaneously to form a helical structure when the extracellular environment becomes acidic [13]. One of the largest advantages of pHLIP is the ability to transport polar and moderately hydrophobic molecules directly into cells, thereby avoiding drug resistance resulting from endocytosis. Many studies have shown that pHLIPs can link a variety of different molecules, such as fluorescent dyes, toxins, drugs, peptides, and peptide nucleic acids (PNAs) [14–21]. Among the pHLIP variants studied, four sequences have high tumor-targeting properties, namely, wild type (WT), variant 3 (Var3), variant 7 (Var7), and ATRAM [22]. One study [19] linked pHLIP (WT) to the P1AP peptide by a non-cleavable linker (chloroacetyl chloride) to obtain pHLIP(WT)-P1AP, which can inhibit the growth of human breast cancer cells (MDA-MB-231 and MCF7 cells) that highly express PAR1 receptor. We made some changes based on a previous study [19], as follows. 1. The vector used in this study was generated with the Var7 sequence of pHLIP. pHLIP(Var7) is the shortest and most polar peptide sequence in the pHLIP family (targeting the tumor), and it has the advantages of easy synthesis, fast blood clearance, etc. pHLIP(Var7) was first applied in a study targeting GPCR. 2. A disulfide bond, instead of chloroacetyl chloride, was used to link pHLIP(Var7) to the P1AP peptide. Disulfide bonds can be lysed in cells; thus, P1AP can be delivered to cells. This method simplifies the synthesis of pHLIP(Var7)-P1AP and is conducive to widespread application.

The cell binding assay in this study showed that (FITC)pHLIP(Var7)-P1AP and MDA-MB-231 cells had high binding ability in an acidic environment (pH 5.0 and pH 6.0); however, at pH 7.4, there was almost no binding, suggesting that pHLIP(Var7) can target acidic tissues, which is consistent with previous studies regarding pHLIP. The antiproliferation assay showed that when the pH was 6.0, pHLIP(Var7)-P1AP significantly inhibited MDA-MB-231 cell growth, suggesting that under acidic conditions, pHLIP(Var7) can effectively deliver the P1AP peptide into MDA-MB-231 cells and that the disulfide bond was cleaved inside the cell and, thus, the P1AP peptide was released into the cells. P1AP played a cytotoxic role by inhibiting PAR1/G protein signal transduction.

Based on previous studies, this study designed and synthesized a novel therapeutic molecule, pHLIP(Var7)-P1AP, that can inhibit the growth of MDA-MB-231 cells. This study confirmed that at the cellular level, pHLIP(Var7)-P1AP can target MDA-MB-231 cells and can effectively inhibit the growth of MDA-MB-231 cells; however, it remains unclear whether MDA-MB-231 solid tumors can be inhibited. This study did not conduct a therapeutic study on animals due to certain restrictions, but future studies will be conducted when conditions permit. We also hope that other researchers can carry out further studies.

## Conclusion

For TNBC, effective treatments are lacking, the prognosis is poor, and the development of new targets has important significance for future treatment strategies. PAR1 is highly expressed on the surface of MDA-MB-231 cells; therefore, PAR1 could be considered a target for the treatment of MDA-MB-231 TNBC. pHLIP(Var7)-P1AP can effectively target MDA-MB-231 cells in an acidic environment and inhibit the growth of MDA-MB-231 cells by inhibiting the signal transduction of PAR1/G protein. pHLIP(Var7)-P1AP is expected to become a new valuable drug for the treatment of TNBC.

## Abbreviations

PAR1: Protease activated receptor 1
pHLIP: PH (low) insertion peptide
TME: Tumor microenvironment
TNBC: Triple-negative breast cancer
